# *NT5C2* novel splicing variant expands the phenotypic spectrum of Spastic Paraplegia (SPG45): case report of a new member of thin corpus callosum SPG-Subgroup

**DOI:** 10.1186/s12881-017-0395-6

**Published:** 2017-03-21

**Authors:** Mahmoud F. Elsaid, Khalid Ibrahim, Nader Chalhoub, Ahmed Elsotouhy, Noora El Mudehki, Alice Abdel Aleem

**Affiliations:** 1 Pediatric Neurology Department, Hamad Medical Corporation, Doha, Qatar; 2 Neurogenetics Research program, Neurology Department, Weill Cornell Medical College, Qatar Foundation- Education City, 24144, Doha, Qatar; 3 Neurology Department, Weill Cornell Medical College, New York, USA; 4 Radiology Department, Hamad Medical Corporation, Doha, Qatar; 5 Physiotherapy Department, Hamad Medical Corporation, Doha, Qatar

**Keywords:** Hereditary spastic paraplegia, SPG-Thin corpus callosum subgroup, Nucleotide metabolism, *NT5C2*, SPG45, SPG45-brain MRI

## Abstract

**Background:**

Hereditary Spastic Paraplegia (HSP) is a genetically heterogeneous group of neurodegenerative diseases. Thin Corpus Callosum (TCC) associated HSP is a distinguished subgroup of complex forms. Purines and pyrimidine, the basic DNA and RNA components, are regulating the cell metabolism, having roles in signal transduction, energy preservation and cellular repair. Genetic defects in nucleotide metabolism related genes have been only recently implicated in brain and neurodegenerative diseases’ pathogenesis.

**Case presentation:**

We present a consanguineous Qatari family with two brothers, 9 and 3 years, who displayed a characteristic phenotype of early onset and markedly-severe spasticity with tiptoe walking, delayed dysarthric speech, persistent truncal hypotonia, and multiple variable-sized areas of brownish skin discoloration appearing at different places on the body. A clinical diagnosis suggestive of complex hereditary spastic paraplegia (HSP) was set after the family had the second affected child. Whole genome sequencing identified a novel homozygous *NT5C2* splice site mutation (NM_012229.4/NM_001134373.2: c.1159 + 1G > T) that recessively segregated in family members. Brain MRI revealed dysgenic and thin corpus callosum (TCC) with peri-trigonal white matter cystic changes in both affected boys, whereas a well-developed corpus callosum with normal white matter was shown in their apparently normal brother, who found to be a carrier for the mutant variant. This mutation led to skipping of exon 14 with removal of 58 amino acid residues at the C-terminal half. The aberrantly spliced NT5C2 showed substantial reduction in expression level in the in-vitro study, indicating marked instability of the mutant NT5C2 protein.

**Conclusion:**

The present report expands the phenotypic spectrum of SPG45 and confirms NT5C2-SPG45 as a member of the rare TCC SPG-subtypes. Homozygous alteration in *NT5C2* seems essential to produce central white matter developmental defects. The study highlights the importance of cytosolic II 5’-nucleotidase (NT5C2) in maintaining the normal balance of purines’ pool in the brain, which seems to play a pivotal role in the normal development of central white matter structures.

**Electronic supplementary material:**

The online version of this article (doi:10.1186/s12881-017-0395-6) contains supplementary material, which is available to authorized users.

## Background

Hereditary spastic paraplegia (HSP) is a large, genetically heterogeneous group of neurodegenerative diseases characterized by retrograde degeneration of axons of motor neurons of the corticospinal tract [1]. A rare subgroup of the HSP recessive-forms was described with a brain-imaging marker of thin corpus callosum (TCC), for which a set of few genes has been identified so far [2]. Genetic defects in nucleotide metabolism related genes have been only recently implicated in HSP pathogenesis [3]. Nucleotidases are a group of hydrolases classified according to their subcellular localization and sites of cleavage. The 5’ nucleotidases catalyze the hydrolysis of 5’ ribo- and deoxyribo-nucleotide monophosphate into the corresponding nucleoside. The 5’ nucleotidase cytosolic II (NT5C2) enzyme has a critical role in maintaining the balance of nucleotides, nucleosides and free nucleobases of purine’s pools in the brain and spinal cord [4]. NT5C2 catalyzes the hydrolysis of Adenosine Monophosphate and Inosine Monophosphate releasing Adenosine. Adenosine has been recently recognized to have a key role in promoting myelin formation in the central nervous system (CNS) [5]. Adenosine inhibits proliferation of oligodendrocytes progenitor cells (OPC), whereas it stimulates their differentiation into mature oligodendrocytes and modulates the communication of the neuron and glial cells with the axons [6–8]. This study describes a consanguineous Qatari family with a provisional clinical diagnosis of complex-HSP, in which the HSP-genes panel testing failed to detect the underlying gene defect. By using whole genome sequencing (WGS) and ingenuity variant data analysis (IVA) we were able to identify a novel homozygous splice site mutation in *NT5C2* that recessively segregates in the family. The *NT5C2* gene involvement in HSP families from Middle East has been reported only once before, however; this is the first report in Qatari patients and demonstrates new clinical findings and interesting observations.

## Cases presentation

### Results

#### Clinical report

Patient II.3 is the youngest (3 years old) of three brothers of consanguineous parents (Fig. 2a). Developmental delay was the first sign that drew the parents’ attention. He remained unable to crawl or sit until the age of 10 months and started tiptoe-walking at the age of 2 years. Progressive spasticity of the lower limbs; limited ankles’ dorsiflexion and hips abduction, with ankle clonus and brisk (+4) reflexes, sitting with bending curved back due to truncal hypotonia, delayed speech and mild mental impairments were his main presentations. His gait was unsteady with knees flexion and lumber lordosis to help to achieve balance.

Patient II.1 is the oldest brother (9 years old). Although he is similarly affected, he was originally misdiagnosed for cerebral palsy because of prematurity and early lower limb spasticity. Postnatally, he was admitted to the NICU because of respiratory distress; however, no mechanical ventilation was required. By the age of 4 months, his lower limbs were markedly spastic with scissoring. As a 2-year-old, his speech and motor developmental delay were notable. He could not sit unsupported for long periods, but could take a few steps while holding onto objects with evident lower limbs spasticity and marked truncal hypotonia. He started to speak when he was 3 years old. On examination, he was alert and cooperative despite delay in active speech. His gait, performed with difficulty for few steps, demonstrated tiptoe walking with limited knee and hip extension and trunk flexion.

At 2 and 3 years old, he was admitted to an inpatient pediatric rehabilitation and training program for 4 months each, which aimed to support motor and speech development. The response was good, as gross motor functions, including sitting, crawling and kneeling, standing, and walking for a short distance without support or orthopedic aid, became possible. He showed a more secure and significantly improved gait, and was able to pick up objects from the ground and walk up to 600 meters without supportive aids. His Gross Motor Function Measure (GMFM) chart, at the age of 3 years showed continued improvements with the percentage of sitting 100%, standing 74% and walking 56%. However, as a 7-year-old, he underwent tenotomies for his spastic hips and ankles.

Follow up at 9 years showed him to have an unsupported gait with primary forefoot contact, knee in flexion and lumber lordosis. Features of truncal hypotonia, markedly limited ankle dorsiflexion and ankle clonus were notable.

His speech showed mild dysarthria. He has learning difficulties and is attending a mainstream school with extra support. Multiple and variable-sized areas of brownish skin discoloration were present at different places on his body (Fig. 1.1).

**Fig. 1:**
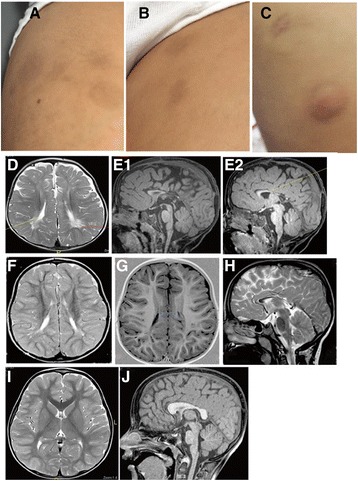
Skin patches and Brain MRI. *1.1*: skin patches of brownish discoloration. Detected in the older patient. Images (**a**, **b**, and **c**) showed multiple, variable-sized areas of darkened “brownish” skin discoloration on the upper right thigh, left thigh and above the right nipple, respectively. The outline-edges of some of those areas look darker. Such variable-sized, multiple and brownish skin areas with darker outline were also seen on the abdomen and upper arms (data not shown). *1.2*: Brain MRI of the three brothers. MRI Scans of younger patient, II.3: Axial T2 weighted image (**d**) showed slit like widely separated lateral ventricles with slightly dilated occipital horns (*yellow arrow*), bilateral peri-trigonal multiple variable size cystic white matter changes, and mild frontal cerebral atrophy. Sagittal T1- weighted images at different levels (**e1**& **e2**) showed markedly hypoplastic corpus callosum with absent splenium and posterior part of its body with secondary uplifted third ventricle. Prominent pre-pontine, supra- and infra- cerebellar cisterns and basal cistern were obviously noticeable in the youngest patient. MRI of older patient, II.1: Axial T2- Weighted Image (**f**) revealed patchy white matter hyperintensities predominantly at the occipital and periventricular white matter. Axial Inversion Recovery Image (**g**) is showing the wide separation of the bodies of the lateral ventricles secondary to hypoplastic corpus callosum. Midline Sagittal T2-weighted image (**h**) displayed the markedly hypoplastic body and absent splenium of the corpus callosum. MRI of Normal brother, II.2**:** Axial T2 weighted (**i**) showed normal white matter volume and signal and normal gyration pattern with well-formed corpus callosum. Midline sagittal T1 weighted image (**j**) is showing the well-developed corpus callosum’ parts

***Brain Magnetic Resonance Imaging (MRI)*** of the two affected boys showed dysgenic/TCC and white matter cystic changes (Fig. 1.2). Images of the unaffected brother (heterozygous for the mutation) showed well-developed Corpus Callosum.

The clinical characteristics of our patients in comparison to other NT5C2-related patients are summarized in Table 1 *.*

**Table 1:** Phenotypic Characteristics in SPG45 patients with NT5C2 mutations

Phenotype Characteristics	Patient II.1	Patient II.3	Families described in Ref.3
Age at onset	7 months	10 months	15 &18 months, infancy, or Unknown
Disease duration	~9 years	~3 years	Range between 2 and 33 years old with a mean of 30 years
Presentation at disease onset	Marked lower limbs spasticity and developmental delay	Developmental delay and marked lower limbs spasticity	Delayed walking
Speech delay	+ (speech started at age 3 years)	+ (speech started at age 2 and half years)	-
Speech dysarthria	+	+	-
Lower limbs Spasticity	Markedly severe	Markedly Severe	Mild
Back hypotonia	+	+	-
Walking ability	Can walk unsupported with abnormal gait	Can walk unsupported with tip toe walking and abnormal gait	Some can walk unsupported, others walk with support
Gait abnormality	+ (Unsteady gait while walking associated with lumber lordosis, knees flexion, limited foot movements, limited knees and hips extension.	+ (Tip toe walking, in-toing, knee flexion (milder than older brother), lordosis, unsteadiness because of movements limitation.	+ (some cases showed associated knees flexion contracture)
Learning disability	+ (attending special school)	+	+ (except in one family reported with normal cognition)
Cerebellar signs	-	-	-
Ophthalmolo-gical signs	Latent convergent Squint improved following correction glasses, optic disk normal	Hyper-metrope, no squint, normal optic disk	Squint, glaucoma, or primary optic atrophy, each sign was reported once in a patient
Brain MRI	TCC, CC dysgenesis, white matter cystic changes and hyperintensities	Similar findings in addition to frontal cerebral atrophy	TCC & white matter changes in 4 families with available images
Skin changes	Variable size patches of brownish discoloration begin to appear at 6 years old	- (he is yet at the age of 3 years)	-
Skeletal abnormalities	Equinus foot	Equinus foot	Equinus foot in one family
Urine incontinence	+ [he developed the control just lately]	+	Nocturnal enuresis in only one case
Reference	This report	This report	[3]

+ : present; − : absent

### Genomic report

**Fig. 2:**
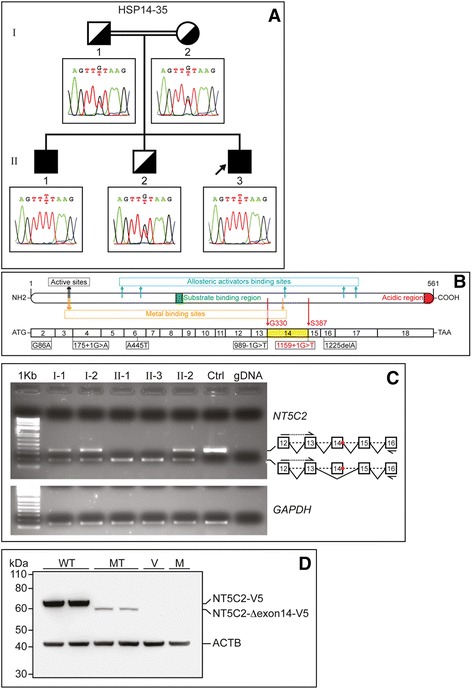
Identification and functional analysis of NT5C2 mutation. **a** Pedigree of the consanguineous family and Sanger sequencing of *NT5C2* splice site mutation. The inheritance pattern is consistent with an autosomal recessively segregated disorder. **b** Schematic illustration of NT5C2 protein and coding transcript. Upper panel, protein structure with depicted active sites (nucleophile and proton donor at positions 52 and 54, respectively), metal binding sites (Magnesium metal at positions 52, 54, 351), allosteric activators binding sites (positions 127, 154, 354, 436, 453), substrate binding region (position 202–210), and Asp/Glu-rich acidic region (position 549–561). G330 and S387 denote boundaries of the deleted region encoded by exon 14. Bottom panel, coding transcript with numbered exons and the location of all the reported *NT5C2* mutations including the present study (*red*). Skipped exon 14 reported herein is highlighted *yellow*. **c** *NT5C2* RNA splicing. RT-PCR using exon14-flanking primers (right diagram depicting exons as *numbered boxes*, introns as *dashed lines*, primers as *half arrows*, and donor splice site mutation as *red circle*) shows the absence of the wild-type (358 bp including exon 14) and the presence only of the mutant (187 bp lacking exon 14) band in the two affected, whereas both bands are present in the heterozygous cases. Results demonstrate skipping of exon 14 from mature *NT5C2* transcript as a consequence of intron 14 donor splice site mutation, without alteration of transcriptional expression. Ctrl is control RNA from unrelated normal individual. Genomic DNA (gDNA) is used to confirm specificity of assay toward spliced RNA. *GAPDH* is housekeeping gene used for normalization. **d** Mutant NT5C2 lacking exon 14 is unstable. In-vitro expression analysis in HEK293 cells transfected with wild type (WT) or mutant (MT) NT5C2 fused to V5 tag. Note the marked low expression levels of mutant NT5C2-Δexon14-V5 (~64 kDa) compared to wild-type NT5C2-V5 (~70 kDa), indicating severe instability of mutant NT5C2 as a consequence of exon 14 skipping. Beta-actin (ACTB) is used as loading control. V, empty expression vector; M, mock; kDa, kilodalton

HSP-panel testing involving 24 known HSP-related genes including those for SPG11 and ﻿SPG15 in a certified clinical lab, did not confirm the clinical diagnosis of HSP and was negative for HSP-related mutations. WGS was performed for five family members; three brothers and the parents. A variant mapped to the consensus donor splice site in intron-14 of Cytosolic-II 5’Nucleotidase (*NT5C2)* on chromosome 10*,* (NM_012229.4/NM_001134373.2, chr10:104852895C > A; c.1159 + 1G > T) was identified and was corroborated by the finding of a homozygosity region on chromosome 10 overlapping with *NT5C2* (Additional file 1: Figure S2). The *NT5C2* variant was not reported in any of the public genome databases; the 1000 genomes project, dbSNP, ESP, and ExAC. And was absent in 108 normal Qatari genomes [9] (Additional file 2: Figure S1). Importantly, *NT5C2* mutations were reported to underlie recessive spastic paraplegia in a previous study [3]; hence, *NT5C2* was our best candidate. Recessive segregation of *NT5C2* variant in the family was confirmed (Fig. 2a). RT-PCR verified the homozygous skipping of exon 14 in the two patients (Fig. 2c). Skipped exon 14 removed 58 residues (G330 to S387) of the 561aa mature protein*;* however, it is in-frame and consequently there was no downstream frameshift. Immunoblots on blood-derived protein lysate of family members revealed no signal; lack of detectable expression of NT5C2 in blood can be a reason (data not shown). The protein expression status of mutant NT5C2 was assessed in vitro by over-expressing both wild type and mutant constructs fused to V5 tag in HEK293 cells. A significant reduction in protein levels of mutant NT5C2 was observed, suggesting altered stability of the mutant form lacking exon-14 (Fig. 2d). Mutant NT5C2 was detectable in-vitro despite its marked reduction, likely due to over-expression experiment; hence, the homozygous splice-mutant NT5C2 was anticipated to be significantly deficient in patient’s tissues and ultimately considered a loss of function mutant.

## Discussion

The NT5C2-related phenotype has previously been clinically assigned to SPG45 (MIM: 613162). This report describes a homozygous novel *NT5C2* splice-site mutation in two Qatari siblings with AR-HSP. This mutation resulted in markedly altered stability of the enzyme-protein. *NT5C2* involvement in recessive HSP was reported once before in a large HSP-cohort study [3]. The splice mutation identified in this study is located at the C-terminal half of the protein and led to in-frame skipping of exon 14. The significantly deficient expression of mutant NT5C2 shown in in-vitro overexpression experiment highlights the substantial impact of exon 14 skipping on protein stability and/or its proper folding; hence a loss of function mutant is assumed.

The six reported *NT5C2* mutations, so far, (3 and this report) (Additional file 3: Table S1) seems to lead to the same path of loss of function. Even so the impact of previously described *NT5C2* mutations on the encoded protein was not tested, however they were predicted to be deleterious and impair the protein function [3].

Phenotypic features of delayed and dysarthric speech, persistent truncal hypotonia, variable-sized patches of skin brownish discoloration and the early-onset, markedly severe spasticity were first described in our patients, expanding the phenotypic spectrum of SPG45.

The skin patches started to appear at 6 years of age; hence it was detectable only in the older patient. There was no clinical reason found for those patches. Altered nucleotide metabolism might be an explanation; follow up with the younger patients might provide a clue.

The capacity to maintain the walking ability despite the marked spasticity (this report) and even in the longest reported disease duration [3, 10] is of a good prognostic value to SPG45 families with NT5C2 mutations.

Studying the brain imaging of the two patients versus the unaffected carrier brother was quite interesting. Brain MRI findings strongly support the observation that homozygous mutation involving the two copies of *NT5C2* is essential to producing the developmental defects in cerebral white matter with dysgenic/TCC. By contrast, the heterozygous status of the mutation in the unaffected brother was associated with normal development of cerebral white matter (Fig. 1.2). Brain images in our cases backed by reports of previously described families with available MRI [3] distinguish SPG45 (*NT5C2*) as an additional member of SPG-TCC subgroup.

Taken together, the mature oligodendrocytes are the main myelin forming cells in the brain and Adenosine is both a modulator of OPC development and a potent neuron-glial-axonal transmitter, it is likely that the normal adenosine and purines’ pool concentrations in the brain has an influence on the normal developmental process of myelin formation in the CNS. Further experiments are necessary to verify this assumption.

We recognized a striking similarity in the key clinical features involving TCC, markedly severe spasticity and long disease duration with maintained ability of unsupported walking in families with NT5C2 (SPG45) or DDHD2 (SPG54) mutations [11, 12]. Mutated phospholipase DDHD2 was suggested to affect both the dynamics and/or morphology of Golgi and ER in a retrograde or anterograde transport mechanism [13, 14] as well as the complex lipid metabolism [15, 16]. Whether there might be a common mechanism potentially linking the NT5C2 loss of function, disturbed purines’ signaling to the transport along the corticospinal tract and/or membrane trafficking, remains a hypothesis awaiting further studies.

## Conclusions

The present report confirms the critical role of cytosolic 5’nucleotidase and nucleotide metabolism in the normal development of central white matter structures and warrants further experiments to explore a potential role of 5’ nucleotidase in transport and/or maintenance along the corticospinal tract. The *NT5C2* c.1159 + 1G > T splicing mutation presents a phenotype of markedly severe and early onset spasticity, persistent truncal hypotonia, delayed-dysarthric speech and skin patches of darkened discoloration. These characteristic features expanding the phenotypic spectrum of SPG45. The present family emphasizes SPG45 with NT5C2 mutations as a member of the TCC-SPG subgroup. Of the notable observation is the good prognosis of the recessive complex SPG45 in terms of mild cognitive impairment with some learning difficulties and the maintained unsupported walking ability, however with abnormal gait.

## Methods

See Additional file 4: Supplementary methods.

## Additional files

Additional file 1: Snapshot of Homozygosity Mapper Analysis of WGS SNPs data. (PDF 491 kb)
Additional file 2: IVA analysis of WGS data. Filters applied (see methods) revealed 3 variants in 3 genes (NT5C2, NINL and KIAA1755) at the genetic analysis filter that showed in IVA to be compatible with Mendelian recessive inheritance (upper panel). (PDF 296 kb)
Additional file 3: NT5C2 mutations reported in NT5C2-associated HSP cases. (DOCX 17 kb)
Additional file 4: Supplementary methods. (DOCX 25 kb)

## Abbreviations

HSP: Hereditary spastic paraplegia
IVA: Ingenuity variant analysis
NT5C2: Nucleotidase 5’ cytosolic II
OPC: Oligodendrocytes progenitor cells
RT-PCR: Reverse transcriptase polymerase reaction
SPG: Spastic paraplegia
TCC: This Corpus Callosum
WGS: whole genome sequencing
